# *N*-[(1-Benzoyl­piperidin-4-yl)meth­yl]benzamide

**DOI:** 10.1107/S1600536814012793

**Published:** 2014-06-14

**Authors:** K. Prathebha, D. Reuben Jonathan, Sathya Shanmugam, G. Usha

**Affiliations:** a PG and Research Department of Physics, Queen Mary’s College, Chennai-4, Tamilnadu, India; b PG and Research Department of Chemistry, Presidency College, Chennai-5, Tamil Nadu, India

## Abstract

In the title compound, C_20_H_22_N_2_O_2_, the piperidine ring adopts a chair conformation. The phenyl rings are inclined to one another by 80.1 (1)° and make dihedral angles of 46.1 (1) and 40.2 (1)° with the mean plane of the piperidine ring. In the crystal, pairs of N—H⋯O hydrogen bonds link the mol­ecules into inversion dimers. C—H⋯O inter­actions further link the mol­ecules, forming a three-dimensional supramolecular network.

## Related literature   

For the synthesis of the title compound, see: Prathebha *et al.* (2013 ▶); Venkatraj *et al.* (2008 ▶). For the biological activity of piperdine derivatives, see: Ramalingan *et al.* (2004 ▶); Sergeant & May (1970 ▶). For bond-length data, see: Allen *et al.* (1987 ▶). For related structures, see: Al-abbasi *et al.* (2010 ▶); Ávila *et al.* (2010 ▶). For puckering parameters, see: Cremer & Pople (1975 ▶).
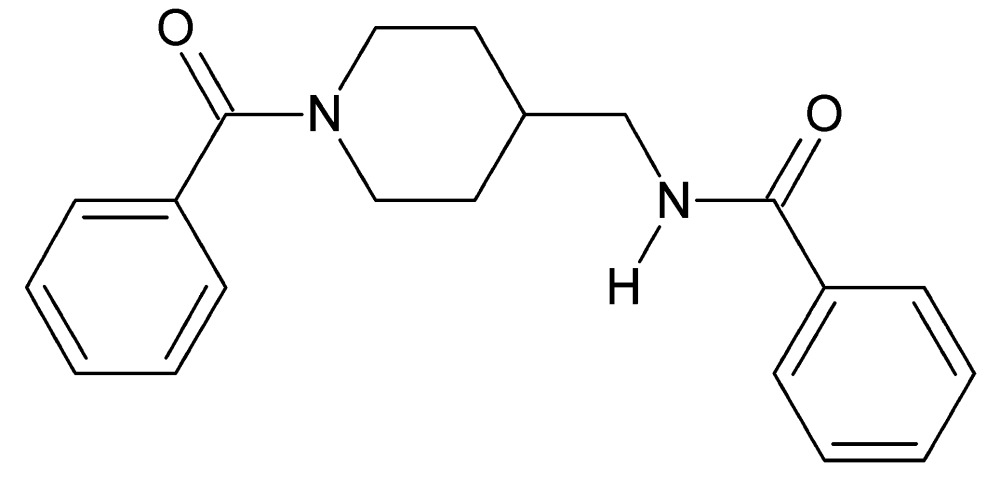


## Experimental   

### 

#### Crystal data  

C_20_H_22_N_2_O_2_
*M* *_r_* = 322.40
Triclinic, 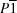

*a* = 9.8039 (2) Å
*b* = 10.4453 (2) Å
*c* = 10.6765 (2) Å
α = 62.208 (1)°
β = 66.009 (1)°
γ = 68.150 (1)°
*V* = 860.80 (3) Å^3^
*Z* = 2
Mo *K*α radiation
μ = 0.08 mm^−1^
*T* = 293 K
0.22 × 0.20 × 0.20 mm

#### Data collection  

Bruker Kappa APEXII CCD diffractometer
Absorption correction: multi-scan (*SADABS*; Bruker, 2004 ▶) *T*_min_ = 0.982, *T*_max_ = 0.984
12912 measured reflections
3562 independent reflections
2929 reflections with *I* > 2σ(*I*)
*R*_int_ = 0.028

#### Refinement  

*R*[*F* ^2^ > 2σ(*F* ^2^)] = 0.043
*wR*(*F* ^2^) = 0.125
*S* = 1.04
3531 reflections
217 parameters
H-atom parameters constrained
Δρ_max_ = 0.56 e Å^−3^
Δρ_min_ = −0.21 e Å^−3^

### 

Data collection: *APEX2* (Bruker, 2004 ▶); cell refinement: *APEX2* and *SAINT* (Bruker, 2004 ▶); data reduction: *SAINT* and *XPREP* (Bruker, 2004 ▶); program(s) used to solve structure: *SIR92* (Altomare *et al.*, 1993 ▶); program(s) used to refine structure: *SHELXL97* (Sheldrick, 2008 ▶); molecular graphics: *ORTEP-3 for Windows* (Farrugia, 2012 ▶); software used to prepare material for publication: *SHELXL97*.

## Supplementary Material

Crystal structure: contains datablock(s) I, New_Global_Publ_Block. DOI: 10.1107/S1600536814012793/bt6968sup1.cif

Structure factors: contains datablock(s) I. DOI: 10.1107/S1600536814012793/bt6968Isup2.hkl

Supporting information file. DOI: 10.1107/S1600536814012793/bt6968Isup3.cml

CCDC reference: 987515

Additional supporting information:  crystallographic information; 3D view; checkCIF report

## Figures and Tables

**Table 1:** Hydrogen-bond geometry (Å, °)

*D*—H⋯*A*	*D*—H	H⋯*A*	*D*⋯*A*	*D*—H⋯*A*
C13—H13*A*⋯O1^i^	0.97	2.60	3.5548 (18)	169
C3—H3⋯O2^ii^	0.93	2.47	3.3803 (17)	167
N2—H2*A*⋯O2^ii^	0.86	2.11	2.9401 (15)	162
C8—H8⋯O1^iii^	0.93	2.52	3.4506 (19)	176

Symmetry codes: (i) 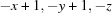; (ii) 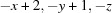; (iii) 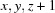.

**Fig. 1.**
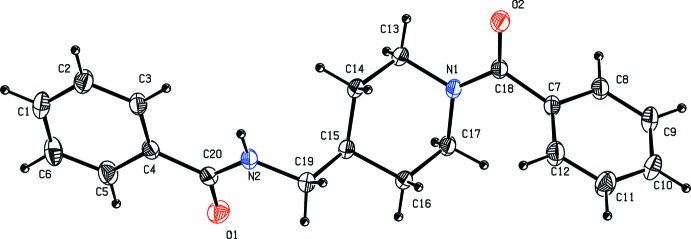
The molecular structure of the title compound, with displacement ellipsoids drawn at the 30% probability level.

**Fig. 2.**
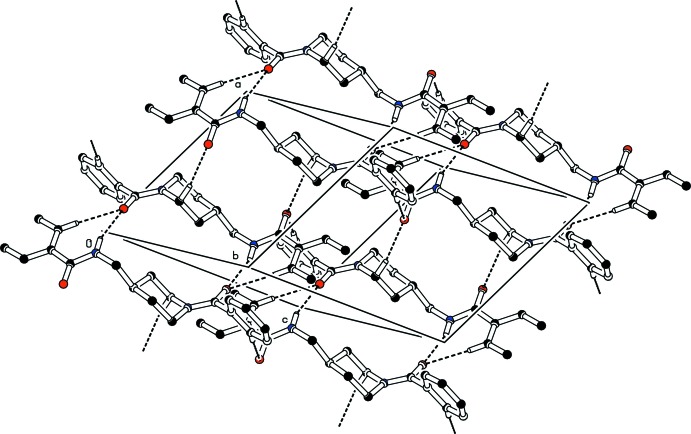
The packing of the molecules in the crystal structure. The dashed lines indicate the hydrogen bonds.

**Fig. 3.**
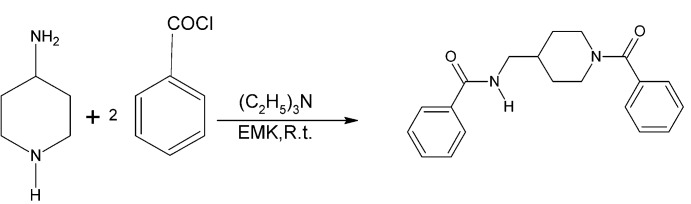
Experimental procedure

## supplementary crystallographic information

### S1. Comment

Biologically active alkaloids of substituted piperidines have been targeted for their total or partial synthesis (Ramalingan *et al.*, 2004). Piperidines are known to have *CNS* depressant action at low dosage levels and stimulant activity with increased doses. In addition, the nucleus also possesses analgesic, anglionic blocking and anesthetic properties as well (Sergeant & May, 1970). We report in this communication, the synthesis and crystal structure of a new piperidine derivative.

The phenyl rings form dihedral angles of 46.1 (1)° and 40.2 (1)°, respectively, with the best plane through the piperidine ring atoms. The C—N distances [1.337 (2)- 1.468 (2) Å] are in the normal range and are in good agreement with values of a similar reported structure (Ávila *et al.*, 2010). The piperdine ring adopts a chair conformation with puckering parameters (Cremer & Pople, 1975) of q2 = 0.0351 (1) Å, phi2 = -50.61 (3)° q3 = 0.5633 (1) Å, QT = 0.5644 (2) Å and θ2 = 3.67 (2)°.

The crystal packing shows N-H···O hydrogen bonds linking the molecules to centrosymmetric dimers (Fig. 2).

### S2. Experimental

The procedure (Prathebha *et al.*, 2013, Venkatraj *et al.*, 2008) adopted in the synthesis of the typical diamide is as follows: In a 250 mL round-bottomed flask 4-methyl piperidine (0.01 mol) was taken in, to which 100 mL of ethyl methyl ketone was added and stirred at room temperature. After 5 minutes, triethylamine (0.02 mol) was added and the mixture was stirred for 15 minutes. Then, benzoyl chloride (0.02 mol) was added and the reaction mixture was stirred at room temperature for about 2 h. A white precipitate of triethyl ammonium chloride was formed. It was filtered and the filterate was evaporated to get the crude product. The crude product was recrystallized twice from ethyl methyl ketone. Melting Point: 127 °C, yield: 85%.

### S3. Refinement

H atoms were positioned geometrically and treated as riding on their parent atoms with C—H = 0.93 - 0.97 Å and N—H. 87 with *U*_iso_(H) = 1.5*U*_eq_ (C-methyl) and = 1.2U _eq_(N,C) for other H atoms.

### Crystal data

**Table:**

C_20_H_22_N_2_O_2_	*Z* = 2
*M**_r_* = 322.40	*F*(000) = 344
Triclinic, *P*1	*D*_x_ = 1.244 Mg m^−^^3^*D*_m_ = 1.188 Mg m^−^^3^*D*_m_ measured by not measured
Hall symbol: -P 1	Mo *K*α radiation, λ = 0.71073 Å
*a* = 9.8039 (2) Å	Cell parameters from 3562 reflections
*b* = 10.4453 (2) Å	θ = 2.3–26.5°
*c* = 10.6765 (2) Å	µ = 0.08 mm^−^^1^
α = 62.208 (1)°	*T* = 293 K
β = 66.009 (1)°	Block, colourless
γ = 68.150 (1)°	0.22 × 0.20 × 0.20 mm
*V* = 860.80 (3) Å^3^	

### Data collection

**Table:**

Bruker Kappa APEXII CCD diffractometer	3562 independent reflections
Radiation source: fine-focus sealed tube	2929 reflections with *I* > 2σ(*I*)
Graphite monochromator	*R*_int_ = 0.028
ω and φ scan	θ_max_ = 26.5°, θ_min_ = 2.3°
Absorption correction: multi-scan (*SADABS*; Bruker, 2004)	*h* = −12→12
*T*_min_ = 0.982, *T*_max_ = 0.984	*k* = −13→13
12912 measured reflections	*l* = −13→13

### Refinement

**Table:**

Refinement on *F*^2^	Primary atom site location: structure-invariant direct methods
Least-squares matrix: full	Secondary atom site location: difference Fourier map
*R*[*F*^2^ > 2σ(*F*^2^)] = 0.043	Hydrogen site location: inferred from neighbouring sites
*wR*(*F*^2^) = 0.125	H-atom parameters constrained
*S* = 1.04	*w* = 1/[σ^2^(*F*_o_^2^) + (0.0625*P*)^2^ + 0.1352*P*] where *P* = (*F*_o_^2^ + 2*F*_c_^2^)/3
3531 reflections	(Δ/σ)_max_ < 0.001
217 parameters	Δρ_max_ = 0.56 e Å^−^^3^
0 restraints	Δρ_min_ = −0.21 e Å^−^^3^

### Special details

**Table:**

Geometry. All e.s.d.'s (except the e.s.d. in the dihedral angle between two l.s. planes) are estimated using the full covariance matrix. The cell e.s.d.'s are taken into account individually in the estimation of e.s.d.'s in distances, angles and torsion angles; correlations between e.s.d.'s in cell parameters are only used when they are defined by crystal symmetry. An approximate (isotropic) treatment of cell e.s.d.'s is used for estimating e.s.d.'s involving l.s. planes.
Refinement. Refinement of *F*^2^ against ALL reflections. The weighted *R*-factor *wR* and goodness of fit *S* are based on *F*^2^, conventional *R*-factors *R* are based on *F*, with *F* set to zero for negative *F*^2^. The threshold expression of *F*^2^ > σ(*F*^2^) is used only for calculating *R*-factors(gt) *etc*. and is not relevant to the choice of reflections for refinement. *R*-factors based on *F*^2^ are statistically about twice as large as those based on *F*, and *R*- factors based on ALL data will be even larger.

### Fractional atomic coordinates and isotropic or equivalent isotropic displacement parameters (Å^2^)

**Table:**

	*x*	*y*	*z*	*U*_iso_*/*U*_eq_	
C1	0.8259 (2)	0.13083 (19)	−0.42562 (19)	0.0716 (5)	
H1	0.8464	0.0451	−0.4428	0.086*	
C2	0.9233 (2)	0.14731 (18)	−0.37349 (19)	0.0687 (4)	
H2	1.0097	0.0725	−0.3554	0.082*	
C3	0.89322 (16)	0.27493 (16)	−0.34793 (16)	0.0557 (3)	
H3	0.9605	0.2865	−0.3147	0.067*	
C4	0.76373 (14)	0.38484 (14)	−0.37167 (13)	0.0462 (3)	
C5	0.6671 (2)	0.3663 (2)	−0.4234 (2)	0.0706 (4)	
H5	0.5791	0.4396	−0.4391	0.085*	
C6	0.6987 (2)	0.2410 (2)	−0.4522 (2)	0.0805 (5)	
H6	0.6338	0.2313	−0.4895	0.097*	
C7	0.75109 (14)	0.87255 (14)	0.19275 (14)	0.0459 (3)	
C8	0.68184 (17)	0.89913 (16)	0.32258 (16)	0.0555 (3)	
H8	0.6521	0.8227	0.4115	0.067*	
C9	0.65695 (19)	1.03945 (18)	0.32005 (18)	0.0655 (4)	
H9	0.6091	1.0571	0.4075	0.079*	
C10	0.7016 (2)	1.15236 (17)	0.1911 (2)	0.0705 (4)	
H10	0.6842	1.2465	0.1904	0.085*	
C11	0.7722 (2)	1.12603 (19)	0.0627 (2)	0.0797 (5)	
H11	0.8044	1.2023	−0.0254	0.096*	
C12	0.7961 (2)	0.98681 (18)	0.06295 (17)	0.0687 (4)	
H12	0.8428	0.9704	−0.0251	0.082*	
C13	0.74162 (16)	0.54026 (13)	0.13846 (15)	0.0491 (3)	
H13A	0.6493	0.5026	0.1824	0.059*	
H13B	0.8142	0.4717	0.1942	0.059*	
C14	0.80986 (15)	0.55186 (13)	−0.02091 (15)	0.0489 (3)	
H14A	0.8293	0.4553	−0.0244	0.059*	
H14B	0.9072	0.5801	−0.0608	0.059*	
C15	0.70474 (15)	0.66528 (13)	−0.11683 (15)	0.0485 (3)	
H15	0.6124	0.6289	−0.0839	0.058*	
C16	0.65720 (17)	0.81395 (14)	−0.09792 (16)	0.0545 (3)	
H16A	0.7453	0.8581	−0.1435	0.065*	
H16B	0.5798	0.8810	−0.1483	0.065*	
C17	0.59429 (16)	0.79586 (15)	0.06342 (16)	0.0535 (3)	
H17A	0.5726	0.8908	0.0712	0.064*	
H17B	0.4992	0.7629	0.1067	0.064*	
C18	0.78445 (14)	0.71670 (14)	0.20002 (14)	0.0455 (3)	
C19	0.78213 (19)	0.68686 (15)	−0.27908 (16)	0.0573 (3)	
H19A	0.7154	0.7658	−0.3369	0.069*	
H19B	0.8762	0.7184	−0.3109	0.069*	
C20	0.71745 (15)	0.52476 (14)	−0.34233 (14)	0.0483 (3)	
N1	0.70509 (12)	0.68746 (11)	0.14379 (12)	0.0470 (3)	
N2	0.81811 (14)	0.55385 (12)	−0.30954 (13)	0.0534 (3)	
H2A	0.9056	0.4929	−0.3064	0.064*	
O1	0.59104 (12)	0.60806 (12)	−0.34789 (13)	0.0697 (3)	
O2	0.88170 (13)	0.62016 (11)	0.25938 (13)	0.0689 (3)	

### Atomic displacement parameters (Å^2^)

**Table:**

	*U*^11^	*U*^22^	*U*^33^	*U*^12^	*U*^13^	*U*^23^
C1	0.0865 (11)	0.0708 (10)	0.0764 (10)	−0.0293 (9)	−0.0096 (9)	−0.0448 (9)
C2	0.0719 (10)	0.0632 (9)	0.0812 (11)	−0.0047 (7)	−0.0229 (8)	−0.0417 (8)
C3	0.0563 (8)	0.0607 (8)	0.0644 (8)	−0.0080 (6)	−0.0217 (6)	−0.0346 (7)
C4	0.0523 (7)	0.0514 (7)	0.0393 (6)	−0.0144 (5)	−0.0127 (5)	−0.0186 (5)
C5	0.0704 (10)	0.0781 (11)	0.0874 (11)	−0.0067 (8)	−0.0393 (9)	−0.0430 (9)
C6	0.0848 (12)	0.0992 (13)	0.0971 (13)	−0.0290 (10)	−0.0304 (10)	−0.0573 (11)
C7	0.0473 (7)	0.0477 (7)	0.0529 (7)	−0.0087 (5)	−0.0160 (5)	−0.0267 (6)
C8	0.0653 (8)	0.0561 (8)	0.0526 (7)	−0.0163 (6)	−0.0129 (6)	−0.0274 (6)
C9	0.0734 (10)	0.0675 (9)	0.0698 (9)	−0.0176 (7)	−0.0082 (8)	−0.0455 (8)
C10	0.0833 (11)	0.0542 (8)	0.0871 (11)	−0.0199 (8)	−0.0168 (9)	−0.0393 (8)
C11	0.1130 (14)	0.0594 (9)	0.0692 (10)	−0.0401 (9)	−0.0106 (10)	−0.0232 (8)
C12	0.0919 (11)	0.0662 (9)	0.0542 (8)	−0.0308 (8)	−0.0042 (8)	−0.0314 (7)
C13	0.0590 (7)	0.0381 (6)	0.0579 (8)	−0.0068 (5)	−0.0231 (6)	−0.0217 (5)
C14	0.0568 (7)	0.0382 (6)	0.0582 (8)	−0.0033 (5)	−0.0214 (6)	−0.0244 (5)
C15	0.0561 (7)	0.0432 (7)	0.0580 (8)	−0.0076 (5)	−0.0229 (6)	−0.0251 (6)
C16	0.0682 (8)	0.0403 (7)	0.0666 (8)	0.0011 (6)	−0.0362 (7)	−0.0249 (6)
C17	0.0558 (7)	0.0474 (7)	0.0720 (9)	0.0045 (6)	−0.0318 (7)	−0.0344 (6)
C18	0.0471 (6)	0.0467 (7)	0.0497 (7)	−0.0062 (5)	−0.0158 (5)	−0.0249 (5)
C19	0.0773 (9)	0.0465 (7)	0.0576 (8)	−0.0108 (6)	−0.0268 (7)	−0.0228 (6)
C20	0.0530 (7)	0.0493 (7)	0.0442 (6)	−0.0080 (6)	−0.0163 (5)	−0.0190 (5)
N1	0.0534 (6)	0.0408 (5)	0.0580 (6)	−0.0019 (4)	−0.0242 (5)	−0.0265 (5)
N2	0.0609 (7)	0.0520 (6)	0.0609 (7)	−0.0032 (5)	−0.0262 (5)	−0.0314 (5)
O1	0.0602 (6)	0.0635 (6)	0.0921 (8)	0.0024 (5)	−0.0320 (6)	−0.0379 (6)
O2	0.0756 (7)	0.0571 (6)	0.0973 (8)	0.0054 (5)	−0.0534 (6)	−0.0382 (6)

### Geometric parameters (Å, º)

**Table:**

C1—C6	1.369 (3)	C13—N1	1.4665 (14)
C1—C2	1.377 (2)	C13—C14	1.5166 (18)
C1—H1	0.9300	C13—H13A	0.9700
C2—C3	1.3854 (19)	C13—H13B	0.9700
C2—H2	0.9300	C14—C15	1.5272 (18)
C3—C4	1.3782 (19)	C14—H14A	0.9700
C3—H3	0.9300	C14—H14B	0.9700
C4—C5	1.3788 (19)	C15—C19	1.5236 (19)
C4—C20	1.5026 (17)	C15—C16	1.5319 (16)
C5—C6	1.378 (2)	C15—H15	0.9800
C5—H5	0.9300	C16—C17	1.517 (2)
C6—H6	0.9300	C16—H16A	0.9700
C7—C12	1.376 (2)	C16—H16B	0.9700
C7—C8	1.3830 (18)	C17—N1	1.4610 (16)
C7—C18	1.5065 (16)	C17—H17A	0.9700
C8—C9	1.3823 (19)	C17—H17B	0.9700
C8—H8	0.9300	C18—O2	1.2277 (15)
C9—C10	1.363 (2)	C18—N1	1.3367 (16)
C9—H9	0.9300	C19—N2	1.4573 (16)
C10—C11	1.369 (2)	C19—H19A	0.9700
C10—H10	0.9300	C19—H19B	0.9700
C11—C12	1.383 (2)	C20—O1	1.2270 (16)
C11—H11	0.9300	C20—N2	1.3387 (17)
C12—H12	0.9300	N2—H2A	0.8600
			
C6—C1—C2	119.84 (14)	C13—C14—C15	112.38 (10)
C6—C1—H1	120.1	C13—C14—H14A	109.1
C2—C1—H1	120.1	C15—C14—H14A	109.1
C1—C2—C3	120.26 (15)	C13—C14—H14B	109.1
C1—C2—H2	119.9	C15—C14—H14B	109.1
C3—C2—H2	119.9	H14A—C14—H14B	107.9
C4—C3—C2	120.12 (13)	C19—C15—C14	111.50 (11)
C4—C3—H3	119.9	C19—C15—C16	109.95 (11)
C2—C3—H3	119.9	C14—C15—C16	109.78 (10)
C3—C4—C5	118.85 (13)	C19—C15—H15	108.5
C3—C4—C20	124.40 (11)	C14—C15—H15	108.5
C5—C4—C20	116.74 (12)	C16—C15—H15	108.5
C6—C5—C4	121.14 (15)	C17—C16—C15	111.99 (11)
C6—C5—H5	119.4	C17—C16—H16A	109.2
C4—C5—H5	119.4	C15—C16—H16A	109.2
C1—C6—C5	119.76 (14)	C17—C16—H16B	109.2
C1—C6—H6	120.1	C15—C16—H16B	109.2
C5—C6—H6	120.1	H16A—C16—H16B	107.9
C12—C7—C8	118.99 (12)	N1—C17—C16	110.21 (10)
C12—C7—C18	122.18 (12)	N1—C17—H17A	109.6
C8—C7—C18	118.70 (12)	C16—C17—H17A	109.6
C9—C8—C7	119.91 (13)	N1—C17—H17B	109.6
C9—C8—H8	120.0	C16—C17—H17B	109.6
C7—C8—H8	120.0	H17A—C17—H17B	108.1
C10—C9—C8	120.86 (14)	O2—C18—N1	122.09 (11)
C10—C9—H9	119.6	O2—C18—C7	119.08 (11)
C8—C9—H9	119.6	N1—C18—C7	118.82 (11)
C9—C10—C11	119.43 (14)	N2—C19—C15	113.75 (11)
C9—C10—H10	120.3	N2—C19—H19A	108.8
C11—C10—H10	120.3	C15—C19—H19A	108.8
C10—C11—C12	120.43 (15)	N2—C19—H19B	108.8
C10—C11—H11	119.8	C15—C19—H19B	108.8
C12—C11—H11	119.8	H19A—C19—H19B	107.7
C7—C12—C11	120.36 (14)	O1—C20—N2	121.84 (12)
C7—C12—H12	119.8	O1—C20—C4	120.44 (12)
C11—C12—H12	119.8	N2—C20—C4	117.72 (11)
N1—C13—C14	109.34 (10)	C18—N1—C17	126.14 (10)
N1—C13—H13A	109.8	C18—N1—C13	120.63 (10)
C14—C13—H13A	109.8	C17—N1—C13	112.60 (9)
N1—C13—H13B	109.8	C20—N2—C19	121.23 (11)
C14—C13—H13B	109.8	C20—N2—H2A	119.4
H13A—C13—H13B	108.3	C19—N2—H2A	119.4
			
C6—C1—C2—C3	−0.1 (3)	C12—C7—C18—O2	107.97 (17)
C1—C2—C3—C4	1.3 (2)	C8—C7—C18—O2	−67.72 (17)
C2—C3—C4—C5	−1.1 (2)	C12—C7—C18—N1	−73.19 (18)
C2—C3—C4—C20	177.72 (13)	C8—C7—C18—N1	111.12 (14)
C3—C4—C5—C6	−0.4 (2)	C14—C15—C19—N2	63.59 (15)
C20—C4—C5—C6	−179.32 (15)	C16—C15—C19—N2	−174.42 (11)
C2—C1—C6—C5	−1.4 (3)	C3—C4—C20—O1	−170.49 (13)
C4—C5—C6—C1	1.7 (3)	C5—C4—C20—O1	8.4 (2)
C12—C7—C8—C9	0.9 (2)	C3—C4—C20—N2	9.15 (19)
C18—C7—C8—C9	176.74 (12)	C5—C4—C20—N2	−172.00 (13)
C7—C8—C9—C10	−0.8 (2)	O2—C18—N1—C17	−176.01 (13)
C8—C9—C10—C11	−0.1 (3)	C7—C18—N1—C17	5.18 (19)
C9—C10—C11—C12	1.0 (3)	O2—C18—N1—C13	−5.8 (2)
C8—C7—C12—C11	0.0 (2)	C7—C18—N1—C13	175.39 (11)
C18—C7—C12—C11	−175.70 (15)	C16—C17—N1—C18	110.77 (14)
C10—C11—C12—C7	−1.0 (3)	C16—C17—N1—C13	−60.11 (14)
N1—C13—C14—C15	−56.11 (14)	C14—C13—N1—C18	−111.16 (13)
C13—C14—C15—C19	174.11 (10)	C14—C13—N1—C17	60.29 (14)
C13—C14—C15—C16	52.02 (15)	O1—C20—N2—C19	−0.2 (2)
C19—C15—C16—C17	−174.15 (11)	C4—C20—N2—C19	−179.78 (11)
C14—C15—C16—C17	−51.14 (15)	C15—C19—N2—C20	89.66 (15)
C15—C16—C17—N1	55.05 (15)		

### Hydrogen-bond geometry (Å, º)

**Table:**

*D*—H···*A*	*D*—H	H···*A*	*D*···*A*	*D*—H···*A*
C13—H13*A*···O1^i^	0.97	2.60	3.5548 (18)	169
C3—H3···O2^ii^	0.93	2.47	3.3803 (17)	167
N2—H2*A*···O2^ii^	0.86	2.11	2.9401 (15)	162
C8—H8···O1^iii^	0.93	2.52	3.4506 (19)	176

Symmetry codes: (i) −*x*+1, −*y*+1, −*z*; (ii) −*x*+2, −*y*+1, −*z*; (iii) *x*, *y*, *z*+1.
